# 16S rRNA assessment of the influence of shading on early-successional biofilms in experimental streams

**DOI:** 10.1093/femsec/fiv129

**Published:** 2015-10-23

**Authors:** Katja Lehmann, Andrew Singer, Michael J. Bowes, Nicola L. Ings, Dawn Field, Thomas Bell

**Affiliations:** 1 NERC Centre for Ecology & Hydrology, Wallingford, OX10 8BB, UK; 2 Queen Mary University of London, London, UK; 3 Imperial College London, Department of Life Sciences, Silwood Park Campus, SL5 7PY, UK

**Keywords:** biofilm diversity, riparian shading, biofilm composition, networks

## Abstract

Elevated nutrient levels can lead to excessive biofilm growth, but reducing nutrient pollution is often challenging. There is therefore interest in developing control measures for biofilm growth in nutrient-rich rivers that could act as complement to direct reductions in nutrient load. Shading of rivers is one option that can mitigate blooms, but few studies have experimentally examined the differences in biofilm communities grown under shaded and unshaded conditions. We investigated the assembly and diversity of biofilm communities using *in situ* mesocosms within the River Thames (UK). Biofilm composition was surveyed by 454 sequencing of 16S amplicons (∼400 bp length covering regions V6/V7). The results confirm the importance of sunlight for biofilm community assembly; a resource that was utilized by a relatively small number of dominant taxa, leading to significantly less diversity than in shaded communities. These differences between unshaded and shaded treatments were either because of differences in resource utilization or loss of diatom-structures as habitats for bacteria. We observed more co-occurrence patterns and network interactions in the shaded communities. This lends further support to the proposal that increased river shading can help mitigate the effects from macronutrient pollution in rivers.

## Abstract (precis)

Riparian shading as a mitigation of harmful algal blooms leads to significant structural changes to both bacterial and algal communities in river biofilms.

## Graphical abstract

**Graphical Abstract Figure.**
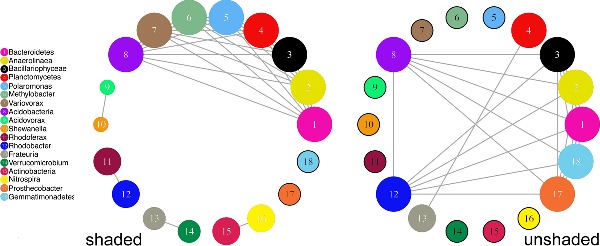
Riparian shading as a mitigation of harmful algal blooms leads to significant structural changes to both bacterial and algal communities in river biofilms.

## INTRODUCTION

Seasonal algal and cyanobacterial blooms have become regular occurrences in many watersheds (Dodds, Smith and Lohman 2002; Paerl, Hall and Calandrino 2011), and are predicted to increase in frequency as a result of human population growth and climate change (Johnson *et al.* 2009; Paerl, Hall and Calandrino 2011). The increasing frequency of algal blooms in rivers worldwide could have substantial economic and ecological consequences, and there is hence much interest in mitigating their impacts. The general consensus is that the primary driver of algal blooms is concentration of macronutrients, which is increasing due to growing human populations, agricultural intensification and increased collection and release of urban wastewater (Mainstone and Parr 2002; Bowes *et al.* 2012b).

Options to reduce harmful blooms have focused on reducing effluent fluxes (Kelly and Wilson 2004; Neal *et al.* 2010). The addition of tertiary treatment to many sewage treatment works has led to substantial reductions in river macronutrient contamination. However, reductions in macronutrient concentration are typically costly and do not always result in reductions in riverine biofilm (or periphyton) growth or in improvements to other proxies of overall river health. What is more, the relationship between harmful algal blooms and macronutrient enrichment is not always a linear one. As Smayda (2008) notes, it is often difficult to trace harmful algal blooms back to nutrient enrichment. In addition, some harmful blooms are caused by nitrogen fixers that thrive under nutrient-limited conditions (Paerl, Hall and Calandrino 2011). In rivers, catchment area, residence time and temperature are also important factors (Desortova and Puncochar 2011; Bowes *et al.* 2012a). Notable examples where nutrient management alone appears to be insufficient to control blooms are the Rivers Thames in the UK and Berounka in the Czech Republic. In both rivers, annual means of soluble reactive phosphorus concentrations have declined over the last decades (from ca 1000 to ca 200 μg l^− 1^ and ca 430 to ca 160 μg l^− 1^, respectively). These concentrations are still high enough for the algal biomass (measured as chlorophyll-*a*) to remain similar to that observed before phosphorus mitigation measures were introduced (Desortova and Puncochar 2011; Bowes *et al.* 2012b). There is therefore a need for complementary controls that enhance the impact of nutrient reductions.

One possibility is to use artificial or natural shading to reduce algae growth rates in catchments with elevated nutrient concentrations. The rationale behind that is that light can be as important in limiting growth of primary producers as macronutrients (Rosemond 1993). Light-limited conditions can also prevent dominance by a few fast growing species, particularly constrain the growth of low-diversity communities consisting of filamentous species that can rapidly take advantage of the high-nutrient environments and create a thick biomass mat that in itself limits the growth of a range of organisms that grow in deeper biofilm layers (Steinman 1992). In support of this, several experiments have shown that light limitation mitigates the impact of eutrophication, even in nutrient rich environments (Triska *et al.* 1983; Hill and Knight 1988; Winterbourn 1990; Hill, Ryon and Schilling 1995; Mosisch, Bunn and Davies 2001; Colijn and Cadée 2003; Hutchins *et al.* 2010; Sanches, *et al.* 2011; Burrell *et al.* 2013). Numerous studies have proposed a range of growth limiting factors for controlling eutrophication. Most often cited is phosphorus; however, just as important is flow velocity, grazing pressure, nitrogen pollution and light (McCall *et al.* 2014). ‘Natural’ experiments have been particularly helpful in elucidating the role of sunlight, whereby increased and decreased periods of natural sunlight on rivers has directly translated into a corresponding increases and decreases in the intensity of the resulting algal bloom (Read *et al.* 2014).

While there have been many studies on the effects of shading on overall measures of biofilm growth, there has been relatively little research on how algal and bacterial biofilm composition is affected by shading. The bacterial biofilm component is less directly affected by light availability, but light levels could affect heterotrophs through changes in UV radiation (Kahn and Wetzel 1999; Yoshikuni 2005; Thomas, Kuehn and Francoeur 2009), or through indirect effects caused by changes to the autotrophic component (Rier and Stevenson 2002). This study uses an experimental approach to compare riverine biofilm communities grown under shaded and unshaded conditions in the River Thames. The Thames catchment is already heavily impacted by anthropogenic activities. High and rising population density in the catchment are projected to put additional pressure on water quality in the Thames, which might be exacerbated by declining river flows and higher water temperatures brought about by climate change (Evans, Spillett and Colquhoun 2003; Neal and Jarvie 2005; EA 2009; Johnson *et al.* 2009). Algal and bacterial biofilm communities were characterized using a molecular approach. Having observed in previous experiments that increased algal growth prompted by excess nutrients lead to biofilms which were dominated by few organisms and were less diverse than those grown under nutrient-limiting conditions, we hypothesized that a similar effect could be observed when light, another strongly limiting factor, was restricted—as high light levels would allow a few fast-growing species to rapidly dominate the communities. We tested two linked hypotheses:

(i) biofilm grown in nutrient rich, shaded conditions assemble significantly different biofilm communities than unshaded communities. Assuming that many members of a biofilm community interact with each other in competitive, predatory or symbiotic ways, any shift in one component of the population would lead to a shift in the others. In that context, we assumed that (ii) the biofilm communities that assemble in the shade are more diverse than those that assemble in unshaded conditions, similar to effects observed in nutrient experiments. The reason for the greater diversity might be due to a reduced abundance of dominant algal species, which lead to a more complex habitat (Bruno, Stachowicz and Bertness 2003).

## METHODS

### Study site

**Figure 1.**
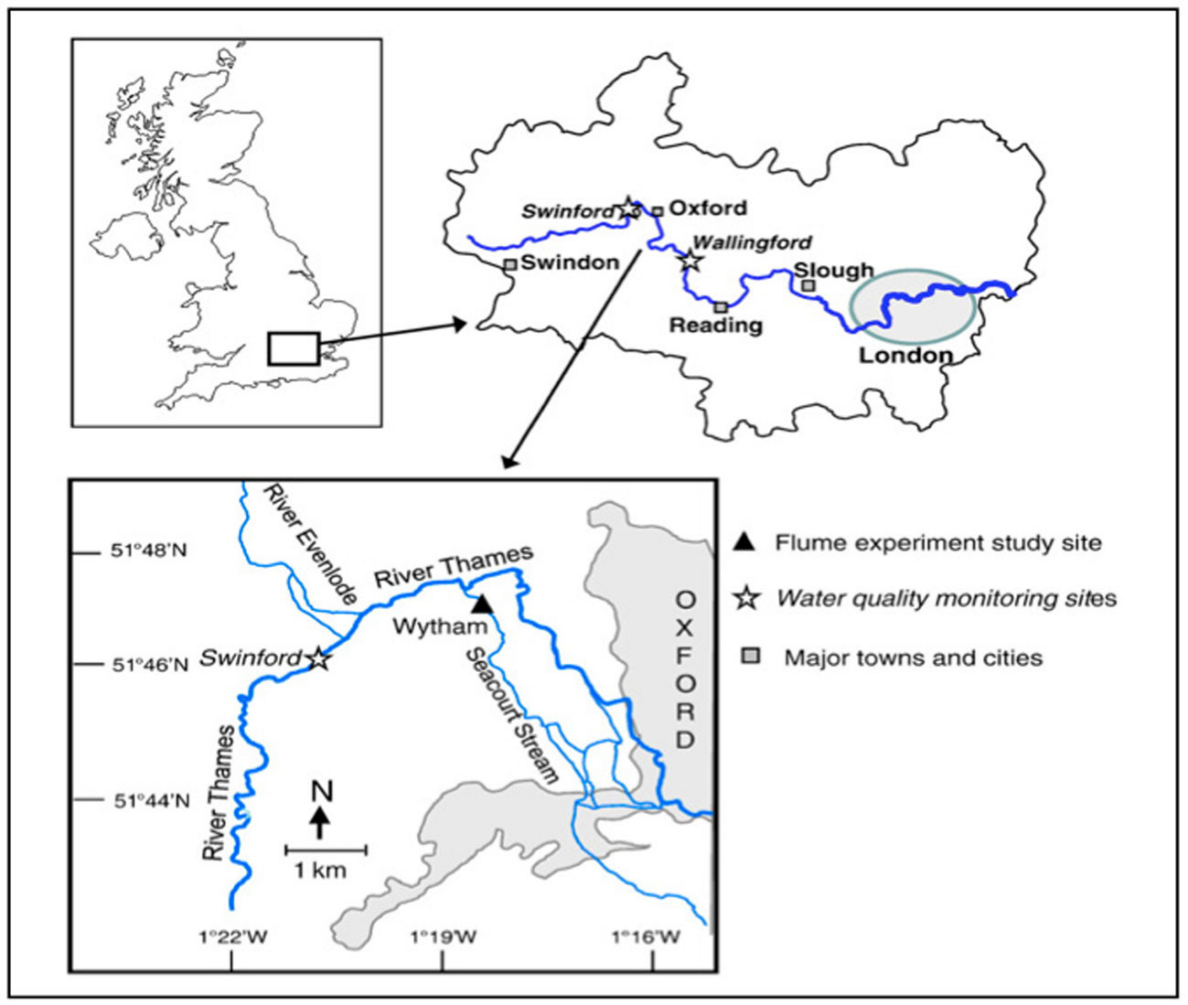
Map of the Thames basin, showing the study location.

The study was conducted in experimental flumes placed within Seacourt Stream, a side-branch of the Thames at Wytham in Oxfordshire, southern England (Fig. 1; 51.786 413 -1.3170 73 Lat/Long, Decimal Geographic Coordinates). Seacourt Stream is a disused millstream directly fed by the Thames (100 m upstream). The site was selected due to its lack of natural shading. Macronutrient composition at the start of the experiment was similar to that in the main Thames branch, and showed little change during the study period (7th–17th September 2010; see results).

### Experiment methodology

**Figure 2.**
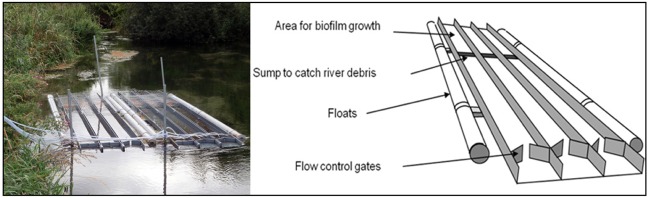
Two mesocosms in the field. The upstream end is closest to the bottom edge of the picture.

We installed 12 experimental flow-through flumes at the study site (Fig. 2). The mesocosms are described in Bowes *et al.* (2010: 384–9). Briefly, they are constructed as blocks of three flumes that float directly in the river, allowing river water to flow through freely. Each flume has a gate to standardize flow rates at the upstream end and a sump to collect river debris $\frac{3}{4}$ of the length from the inlet (Bowes *et al.* 2010). The flumes are made of polyvinyl chloride sheeting set in an aluminium frame, with each flume measuring 5 × 0.3 m. For this experiment, four sets of flumes (i.e. 12 flumes in total) were tethered to the riverbank and positioned 0.5 m above the streambed, held by scaffolding poles. Floats ensured a constant water depth of ∼6 cm inside the flumes. The gap between the riverbed and mesocosms limits invertebrate colonizers from entering the flumes.

Before the start of the experiment, we measured midday light intensity with a SunScan SS1 light probe in both direct sunlight and full tree shade. In full tree shade, the intensity of direct sunlight was reduced by 71%. We used layers of greenhouse shading mesh, positioned directly above the flumes, to create light intensities equivalent to those measured in full tree shade over parts of the flumes. Dividing each channel in half, we grew shaded and unshaded biofilm next to each. A similar approach has been used in other studies investigating the effect of light on biofilm (Rier, Stevenson and LaLiberte 2006; Hill, Fanta and Roberts 2009; Hill *et al.* 2011). We positioned temperature loggers (iButton, Maxim, San Jose, CA USA) next to each set of tiles to determine whether the shading also reduced water temperature. We set the flow rates in each channel to 0.1 m s^−1^ (measured using a Valeport 801 flow meter) at the start of the experiment.

### Sample collection and DNA sequencing

We grew biofilm on 2 × 2 cm limestone tiles anchored to the bottom of the channels for 9 days. This might have lead to a community that was not fully mature, but when left longer, biofilms in previous experiment invariable sloughed off the tiles and floated downstream. In the upstream half of each flume, there were three shaded tiles and three unshaded tiles. On day 9, we harvested the biofilms in all flumes and extracted the DNA, pooling the three tiles within each treatment/flume. Briefly, we added 300 μl of lysis buffer [100 mM NaCl, 500 mM Tris (pH 8), 10% (w/v) sodium dodecyl sulfate, 2 mg ml^−1^ proteinase K, 2 mg ml^−1^ lysing enzyme mix (both Sigma-Genosys, Gillingham, UK)] and 300 μl of NaH_2_PO_4_ (pH 8.0) to the pooled sample, incubated the DNA in a 55°C water bath for 30 min and mixed every 10 min., added 80 μl of prewarmed 10% CTAB solution (65°C), incubated in 65°C for 10 min, added 680μl chloroform:isoamyl alcohol (24:1 vol/vol). The tubes were centrifuged for 5 min at 14 000 rpm. The aqueous top layer was aspirated into a new tube and the DNA precipitated by adding 300% (w/v) TE Buffer, pH 8.0 (10 mM TRIS-HCl, 1 mM EDTA, pH 8.0) and 200% (w/v) PEG/MgCl_2_ mix (30% (w/v) PEG 8000, 30 mM MgCl_2_), leaving the samples overnight at 5°C (Paithankar and Prasad 1991). We then centrifuged the replicates (12 per treatment) for 10 min at 14 000 rpm, discarded the supernatant and washed the DNA pellets by adding 300 μl 70% chilled ethanol. We centrifuged the tubes again, discarded the ethanol and left the tubes to dry in a laminar flow cabinet until the ethanol had evaporated. We added 50 μl ultrapure water and left the DNA to resuspend for 1 h on the bench. We used the 454 GS-FLX TITANIUM platform (Roche 454 Life Sciences, Branford, CT USA) to produce tag-encoded 16S amplicons of ∼400 bp length. We targeted a fragment of the 16S ribosomal RNA (rRNA), comprising the V6 and V7 regions. Primers used for the PCR were 967F, 5′-CNACGCGAAGAACCTTANC-3′ and 1391R, 5′- GACGGGCGGTGTGTRCA-3′ (Huse *et al.* 2008; Huber *et al.* 2009: 1292–302). These universal primers are designed to amplify a large variety of 16S sequences, but, as with all universal primers, it cannot be excluded that some OTUs (both chloroplast and bacterial 16S rRNA) did not get captured. The sequencing libraries were generated through a one-step PCR with a total of 30 cycles, a mixture of Hot Start and Hot Start high fidelity taq polymerases and amplicons extending from the forward primers. DNA amplification and pyrosequencing were carried out at Research and Testing Laboratory (Lubbock, TX USA).

### Bioinformatics

We used CloVR 1.0 RC4 (Angiuoli *et al.* 2011) on the Data Intensive Academic Grid (DIAG, University of Maryland, USA) to run the QIIME workflow ‘pick_otus_through_otu_tables.py’ (Caporaso *et al.* 2010). Within the QIIME workflow: (i) we set the minimum and maximum sequence length to 100 and 2000 bp, respectively, the maximum homopolymer length to 8 bp and maximum number of ambiguous calls to zero; (ii) clustering was performed using UCLUST with a nucleotide sequence identity threshold within each cluster at 97% and alignment against the Greengenes 16S database with PyNAST; (iii) taxonomy assignment of each OTU-representing sequence through the RDP classifier with a confidence threshold of 0.8. After quality control, the data set consisted of 101 617 combined reads for all 12 flume channels of the experiment. Clustering and chimera removal left 97 065 combined reads. Following from earlier studies (Pillet, de Vargas and Pawlowski 2011; Lindemann *et al.* 2013), we used the chloroplast 16S rRNA to focus on the algal communities. Therefore, of those OTU's that were identified to Genus level, we divided the community into algal-derived chloroplast reads and bacterial (including cyanobacterial) reads. We equilibrated the number of sequences per sample by randomly sampling without replacement (Hamady and Knight 2009; Koren *et al.* 2013), resulting in 290 algal sequences per sample, and 732 bacterial sequences per sample. OTUs that are discussed on the species level were blasted individually against the RDP database (Cole *et al.* 2009). Only fragments that could be matched at 97% or above were classified to species level. The rarefied OTU tables were imported into Primer (PRIMER-E Ltd, Ivybridge, UK), MEGAN (Huson *et al.* 2007) and R for further analysis.

### Statistics

We were interested in how shading altered biofilm community composition and diversity. We calculated diversity, tested that the diversity data was normally distributed, then compared diversity across the treatments using analysis of variance (ANOVA). We included both treatment and channel as factors. We added the location of the flumes within the river channel as an additional factor/error, because not all flumes could be placed next to each in the river channel and location effects could not be excluded. We compared dissimilarity in community composition by calculating Bray–Curtis dissimilarities between pairs of communities (Bray and Curtis 1957). We tested for differences between treatments using PERMANOVA, a multivariate permutation test analogous to ANOVA (Anderson 2005). The PERMANOVA design was two-factorial, including treatment and channel. We used non-metric multidimensional scaling (NMDS) (Kruskal 1964) to visualize differences between the communities. We then used similarity percentages (SIMPER) to explore the contribution of each species (Clarke 1993). SIMPER assesses the contribution of each species to the observed dissimilarity between communities. PCR-based data cannot be used to accurately assess relative abundances in the original samples, but given that all samples were amplified in the same way, and we assume that abundances can be compared between our samples (but not with samples from other datasets). Lastly, we used network analysis of co-occurrence patterns (Barberan *et al.* 2012) to explore possible connections between biofilm components. We used network analysis implemented in the MEGAN software package (Huson *et al.* 2007) to visualize co-occurrence patterns in our data. The visualization connects OTUs (here at the taxonomic level of Class) that exceed a prescribed probability of co-occurrence. We set the following threshold values: the threshold required for a taxon to be considered present in a sample was 0.5%; the minimum and maximum percentage of samples in which a taxon could occur was set to 15% (2 samples) and 100% (12 samples); the minimum probability that a co-occurrence between two taxa had been observed was set to 95%. All of the statistical results and figures were produced using CloVR (Angiuoli *et al.* 2011), MEGAN or the R environment (www.r-project.org). Significance thresholds of *P* < 0.05 were used throughout to validate the results.

## RESULTS

**Figure 3.**
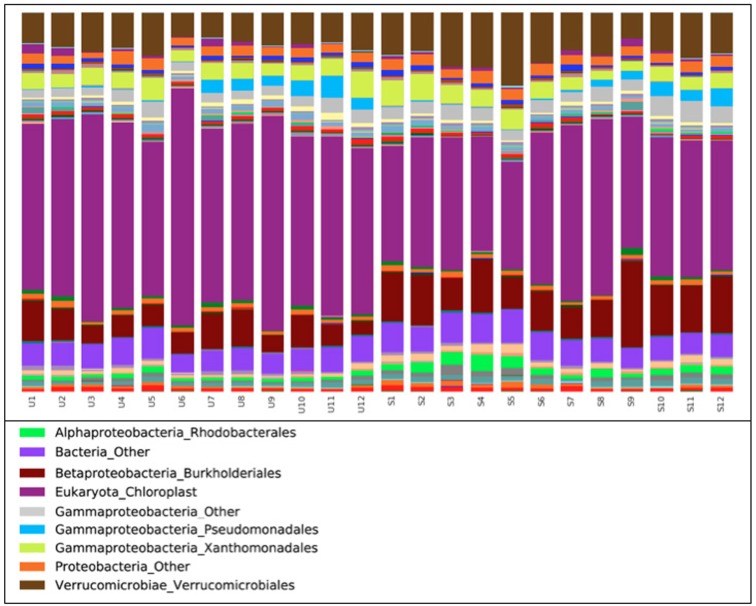
Taxonomic distribution chart of all replicates at order level, based on relative abundance of 16S OTUs from the pyrosequencing results. Algae did not get resolved at order level. The most abundant organisms are listed in the legend. The *x*-axis categories show the replicates: U1–U12 are the unshaded samples, S1–S12 the shaded samples. Bacteria_Other defines the group of bacterial organisms that cannot be identified to other taxonomic levels.

**Figure 4.**
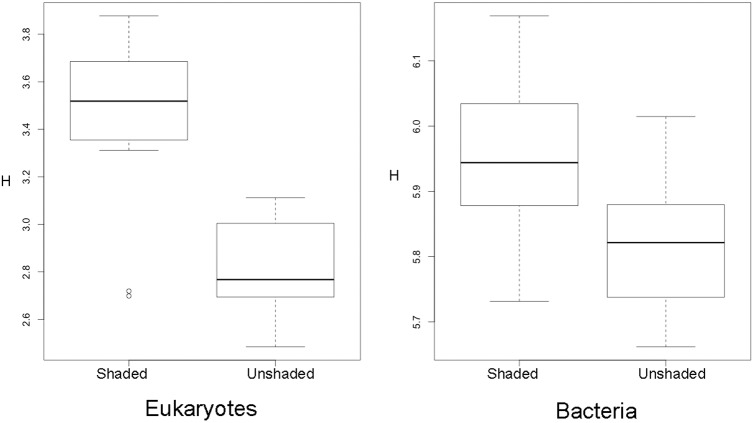
Shannon diversity of both the algal and bacterial components of the biofilm samples. ANOVA: *F* = 32.70 (Algae) and 9.168 (Bacteria), *P* = 1.35e-05 (Algae) and 0.007 (Bacteria).

**Figure 5.**
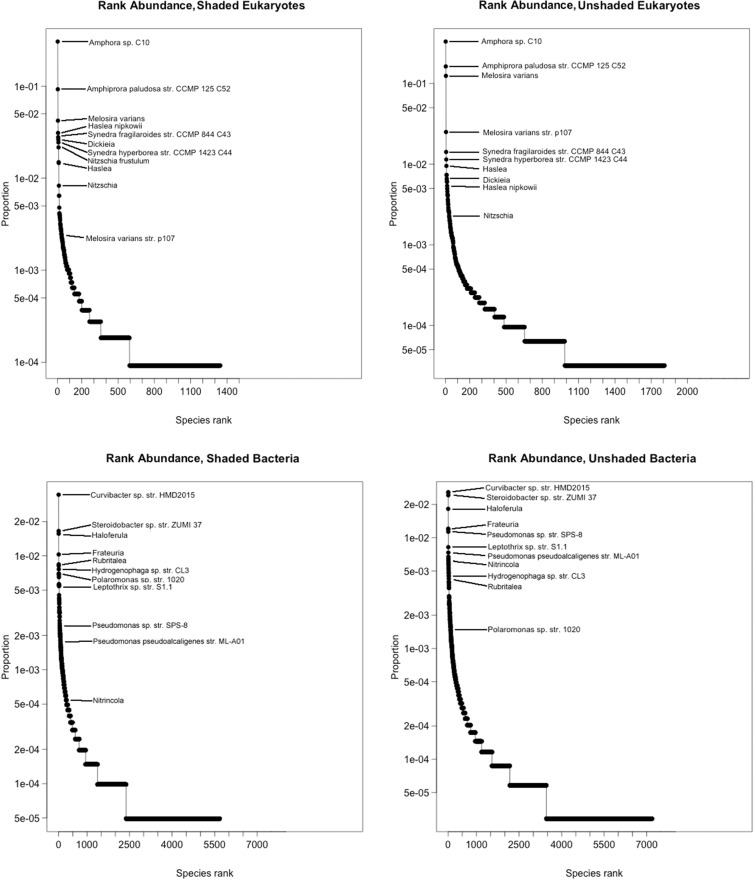
Log-transformed rank-abundance curves for both shaded and unshaded algae and prokaryotes. Species that rank high in the Simper analysis are marked by name.

The taxonomic distribution of the data shows that a large proportion of taxa were of algal origin (Fig. 3). A total number of 19% of sequences could not be identified to Genus level, but only 1% of the 44% that were identified as algae were not identifiable to Genus level. The Shannon diversity (Fig. 4) of both the algal and bacterial communities was significantly higher in the shaded communities [*F*_1,1_ = 36.4 (Algae) and 7.1 (Bacteria), *P* = 1.26e-04 (Algae) and 0.02 (Bacteria)]. In the algal component of the biofilm, the community was dominated by *Amphora* sp. *C10*, *Melosira varians* and *Amphiprora paludosa* str*. CCMP 125 C52*, which accounted for 62% of overall relative abundance (Fig. 5). In contrast, in the shaded community these three species were still dominant but accounted for only 44% of the overall relative abundance (Fig. 5). For the bacterial component of the unshaded communities, the most abundant species were *Curvibacter* sp. str*. HMD2015* (2.4%) and *Steroidobacter* sp. str*. ZUMI 37* (2.4%). These two species were also the most abundant in the shaded community (3.4 and 1.7%), but in both cases they accounted for only approximately 5% of the total community (Fig. 5). Applying Pielou's evenness measure to the samples confirms that the bacterial components of the biofilm were significantly more even in composition than the algal ones, in both the unshaded and shaded replicates (0.95 for the bacterial components, 0.7 for the algal component, *F* = 460, *P* < 2e-16).

**Figure 6.**
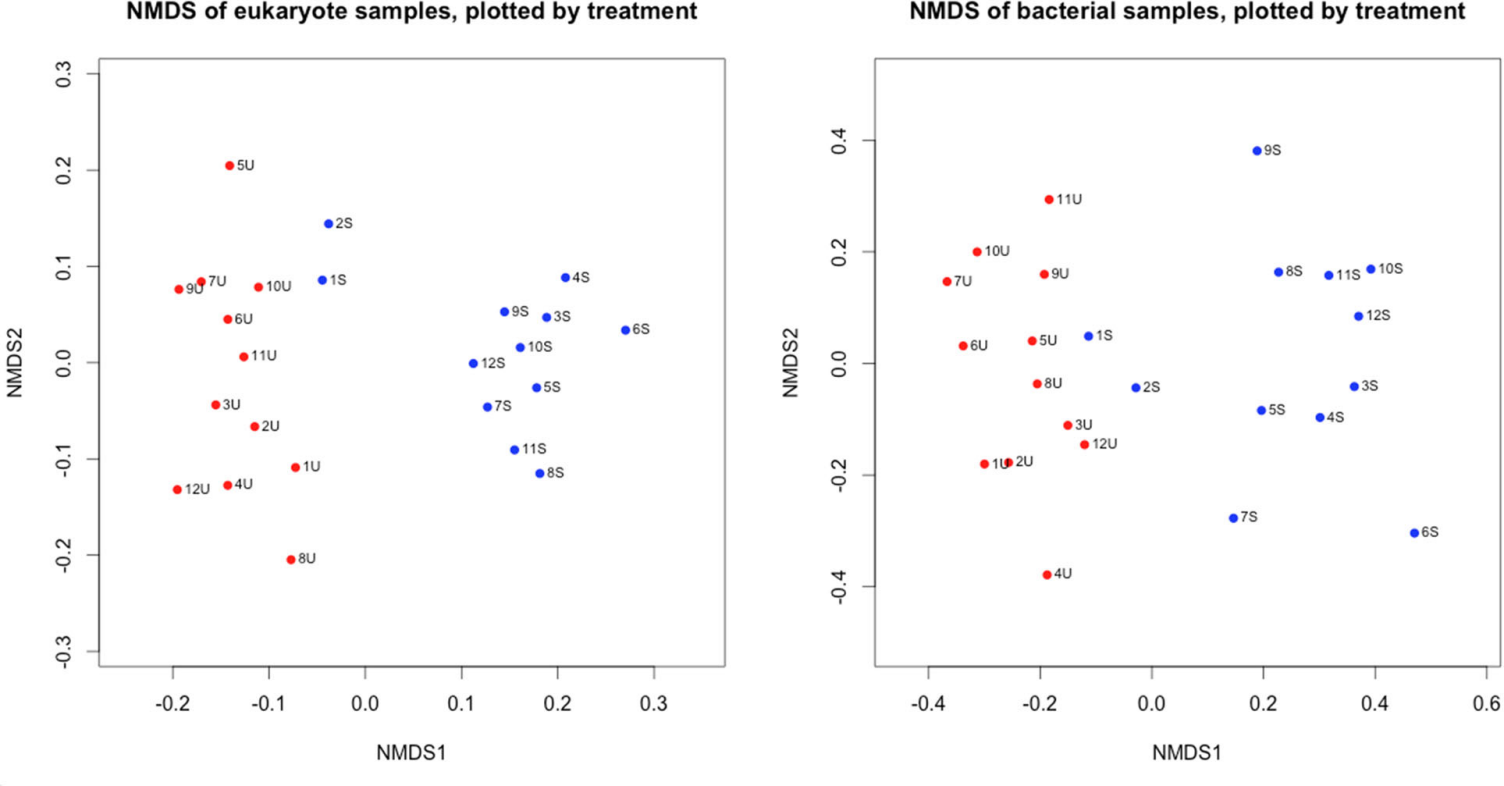
NMDS of the shaded (S) and unshaded (U) replicates, divided into algae and prokaryotes, from Seacourt Stream. Stress: 0.19 (Algae)/ 0.18 (Bacteria).

Ordination of the communities using NMDS (Fig. 6) indicates distinct clusters of unshaded and shaded communities for both the eukaryotes and bacteria (PERMANOVA: eukaryotes, Pseudo *F* = 7.60, *P* = 0.002 and bacteria, Pseudo *F* = 2.52, *P* = 0.006). The unshaded communities are less variable than the shaded ones.

We used SIMPER analysis (Clarke 1993) to investigate which OTUs contributed most to the observed dissimilarity between the shaded and unshaded assemblages. In the algal component, the three most abundant diatoms also contributed most to the observed dissimilarity: *Amphora* sp. *C10* contributed 8.6%, *Melosira varians* 8.4% and *Amphiprora paludosa* str. *CCMP 125 C52* 6.5%. All three were more abundant in the unshaded treatment and together accounted for 23% of the overall observed difference. The next six important algal contributors, however, were more abundant in the shaded treatment than in the unshaded treatment, and accounted for 14% of the overall observed difference. Due to the greater evenness of the bacterial biofilm component, the 10 major contributors in the bacterial replicates only accounted for 6% of the overall dissimilarity, with the two most abundant species (*Steroidobacter* sp. str*. ZUMI 37* and *Curvibacter* sp. str*. HMD2015*) contributing just 2% of dissimilarity.

**Figure 7.**
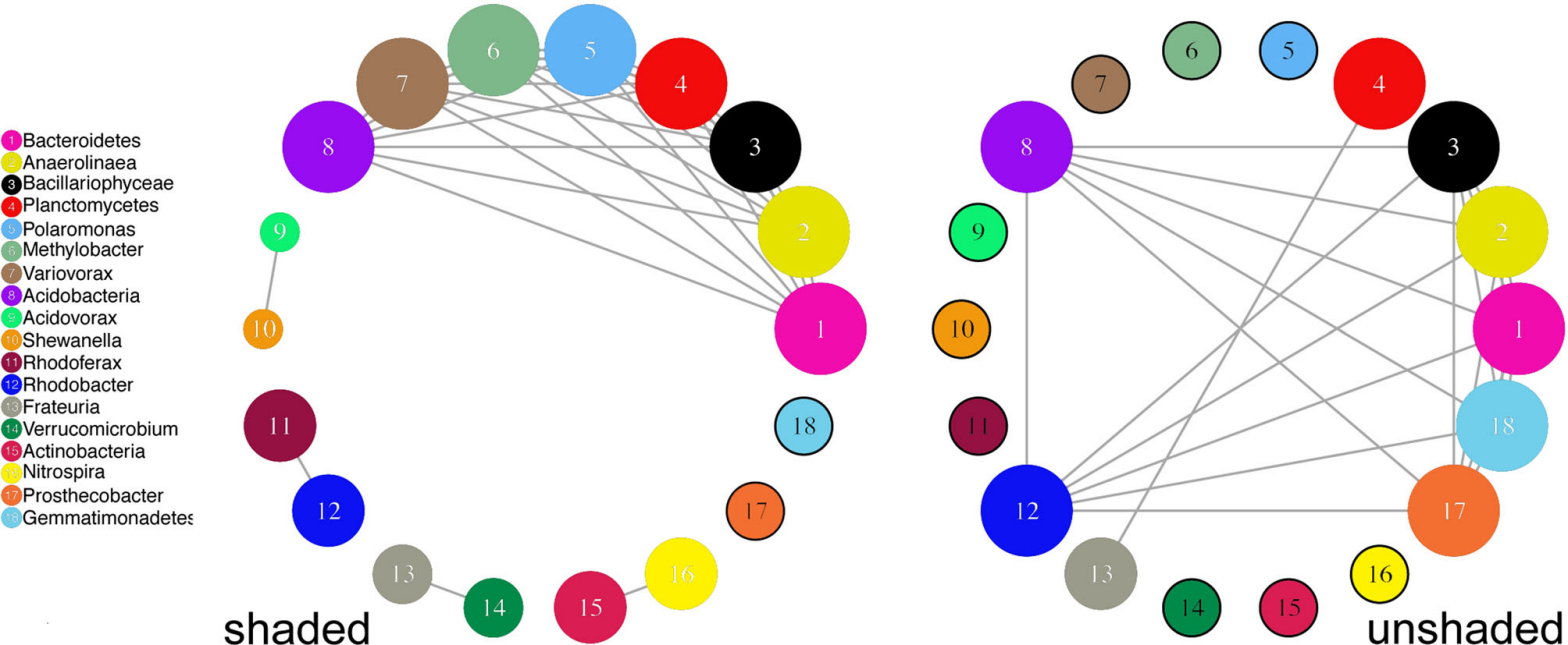
Networks of co-occurrence. Threshold: 0.5%, minimum/maximum:15%(2 samples)/100%(12 samples). Minimum probability that a co-occurrence between two taxa can be observed: 95%.

We used co-occurrence network analysis to explore the relationship between bacterial and algal taxa (Fig. 7). The figure shows one main network in the unshaded samples, consisting of common bacteria and diatoms found in all twelve replicates (Fig. 7; *Bacillariophyceae, Gemmatimonadetes, Rhodobacter, Bacteroidetes, Prosthecobacter, Acidobacteria, Anaerolineae*). This major network was also detectable in the shaded replicates, consisting of four of the nodes that were present in the unshaded samples (Fig. 7; *Bacillariophyceae, Bacteroidetes, Acidobacteria, Anaerolineae*). These were joined by four additional nodes, differing from the ones in the unshaded samples (Fig 7: *Methylobacter, Variovorax, Polaromonas, Planctomycetes*). That means this network included one additional member in all twelve replicates of the shaded treatment.

In eleven of the unshaded replicates, there was also a small network consisting of just two nodes, *Frateuria* and *Planctomycetes*. This 2-node-network containing *Frateuria* was also detectable in ten of the shaded samples, but the *Planctomycetes* node was replaced by *Verrucomicrobia*.

In eleven of the shaded samples, there were also two small networks that were absent in the unshaded samples (*Actinobacteria* and *Nitrospira, Rhodobacter* and *Rhodoferax*), and in two of the replicates there was a network consisting of *Acidovorax* and *Shewanella*.

Throughout the experiment, Seacourt Stream had nutrient concentrations of around 234 μg l^− 1^ soluble reactive phosphorus (SRP), 5.18 mg l^−1^ nitrate (N), and the dissolved reactive silicon 3.05 mg l^−1^, which is considered high for SRP, low for N, and below average for silicon (Neal *et al.* 2005; EA 2012). Silicon is typically depleted at periods when diatoms are ‘in bloom’, which is consistent with this period of study. The water temperature of the shaded areas was at all times identical to that of the unshaded areas and in all channels (averaging at 15.5ºC) throughout the experiment.

## DISCUSSION

This study joins a number of other studies in observing significant changes to algal assemblages under reduced light conditions (O'Driscoll, Harrison and Giller 2006; Guariento *et al.* 2011; Bowes *et al.* 2012b). Uniquely, however, our results show that reduced light conditions affect both algal and bacterial components of biofilm.

A marked result of shading is a change of dominance in the most prevalent organisms. Whilst PCR can skew abundance patterns found in the original sample, the decrease in dominance of the three most abundant diatoms, *Amphora* sp. *C10, Melosira varians* and *Amphiprora paludosa* str. *CCMP 125 C52*, under shaded conditions is consistent with findings by Hill *et al.* (2011), who note in addition, that light is a more limiting factor for autotrophs than nutrient availability. In another study, Sanches *et al.* (2011) confirm and expand on these findings by showing that low light availability does not only limit biofilm growth, but also nutrient propagation within the biofilm, thereby affecting the autotrophic to total biofilm biomass ratio. In the Sanches *et al.* (2011) experiment, autotrophic biomass was highest under high light conditions and N enrichment, whereas heterotrophic biomass increased under enrichment for both P and N (Sanches *et al.* 2011). To relate that back to the Seacourt Stream experiment: the macronutrient content in Seacourt Stream measured at the beginning of the experiment was sufficiently enriched enough to allow for an increase in abundance of autotrophs. We have shown this increase in a parallel experiment, which measured biomass and chlorophyll a content conducted in the downstream area of the same flumes (published as Bowes *et al*. 2012b). The parallel experiment certainly showed higher algal biomass accrual in the unshaded treatment, and that is probably reflected in the observed increase of the dominant diatom. Results from Bowes *et al.* (2012b) further support the findings of this study in as much as the biomass on tiles of the shaded treatment showed greater algal diversity than the unshaded tiles.

The changes in the bacterial community components were less marked than for the algae, but again the most visible pattern was a reduction in the relative abundance of the most prominent organisms in the shaded samples. One potential explanation is that this is driven partly by the correlated changes in the algal community. A possible reason for this is that specific bacteria utilize organic compounds excreted by algal species (Ylla *et al.* 2009). A greater diversity of algae might provide a higher diversity of exudates, which would in turn support a higher diversity of heterotrophs. Kritzberg, Langenheder and Lindstrom (2006) have stated that 30–65% of bacterial production in lakes is supported by algae-derived autochthonous carbon. A reduction in the biomass of algae due to less light is also likely to have lead to a reduction in autochthonous carbon availability on the tiles, to which the bacterial community responded with a shift in structure. Chang (2010) has hypothesized that such structural changes could be due to a shift from heterotrophic consumers, to consumers that are more likely adapted to allochthonous carbon sources. Finally, many bacteria colonize the exoskeletons of diatoms and their diffusive boundary layer, a thin layer of fluid directly surrounding the diatom known as the ‘phycosphere’ (Bell and Mitchell 1972; Rier and Stevenson 2002; Znachor, Simek and Nedoma 2012). This layer contains extracellular products generated by the diatom, and bacteria living in the vicinity might provide the diatom with products it cannot produce itself (Amin, Parker and Armbrust 2012). A reduction of *Bacillariophyceae* numbers would also reduce the number of such colonizers. The co-occurrence network analysis suggests that there were indeed a number of bacterial classes in our samples with occurrence patterns that matched those of the *Bacillariophyceae* in the experiment.

It is not possible to infer much about the nature of taxon interactions from our data, but the result of the co-occurrence analysis could suggest that the influence of light is so great, that interactions between taxa are less important when enough light is available. To expand on that, under shaded conditions, a reduced amount of freely available DOM and other metabolites might make it more important for biofilm organisms to interact with cohabitants of the biofilm, as possibly indicated by the increase of co-occurrence networks in the shaded replicates. At the same time, light limitation might forge relationships that differ from those in light non-limited conditions, as seen in the changing *Frateuria* networks. A possible cause for the network formations observed between *Frateuria* and other organisms could be that *Frateuria* is unable to synthesize some of the compounds required for its growth (Hashidoko 2005). In the unshaded samples, *Frateuria* appears to form a network with *Planctomycetes*, but in the shaded samples, *Planctomycetes* are part of the network with most nodes, whereas *Frateuria* forms a separate network with *Verrucomicrobia.*

The *Verrucomicrobia* are found in a greater number of the shaded samples than the unshaded samples, but it is probably more relevant that in the shaded samples, the *Planctomycetes* are part of the main network. One possible cause for this is that the shaded and unshaded replicates harbor different species of *Planctomycetes* and *Verrucomicrobia*. Another cause could, however, be that in the shaded samples, the *Planctomycetes* rely on a close relationship with other organisms to obtain products, which under non-limited light conditions, are easily available, leaving *Frateuria* to form relationships with alternative organisms. Any statement on the nature of the exchanges is purely speculative, but it may be interesting to note that *Frateuria* have been linked to methanogens in the past (Romanovskaya and Titov 1992), and that both *Verrucomibrobia* and *Planctomycetes* include methanogens amongst their groups of species (Chistoserdova *et al.* 2004; Dunfield *et al.* 2007). A possible outcome of such a change in interactions could be that there are functional differences between shaded and unshaded communities.

All of the dominant diatom species in this study can cause blooms (Hillebrand and Sommer 1997; Ohtsuka 2005; Khare and Chaurasia 2009; Vanelslander *et al.* 2009; Dorigo *et al.* 2010; Paerl, Hall and Calandrino 2011). Even though we observed that a shaded environment led to less dominance of diatom taxa, it cannot be excluded that these diatom taxa could adjust to shady conditions by reaching saturation levels at lower light intensities, as previously described (Rier, Stevenson and LaLiberte 2006). Notably, there were two possible blooming species, *Haslea nipkowii* and *Synedra hyperborea* str. *CCMP* that were more abundant under shaded than unshaded conditions. Likewise, 15 of the 29 observed cyanobacteria species were more abundant under shaded conditions, too. This means that riparian shading can only be one tool in managing algal and cyanobacterial blooms. Lastly, it is important to consider what effect riparian shade has on other riverine organisms. Invertebrates, for example, have been shown to decline when shading is increased by more than 60% (Quinn *et al.* 1997). Hence, mitigating measures for ‘algal’ blooms, such as shading, could have wider ecosystem implications.

There was a surprisingly low abundance of algal genera other than diatoms. It is possible that the experiment was stopped before a significant number of filamentous algae could establish themselves. Inferences drawn in this study might therefore only be applicable to early succession biofilms. Alternatively, the low number of filamentous species could have been the result of the exclusion of snails from the flumes, which selected for diatoms (Rosemond 1993). It is also unclear whether the universal primers that were used to amplify our 16S sequences might have been more suitable for diatoms than for filamentous algae (Chung and Staub 2003). The shaded replicates had two outliers (S1and S2), which probably received more sun during the experiment due to the sun's angle at particular times of the day; however, these did not affect the statistical significance of the overall results.

## CONCLUSIONS

Shading has a marked effect on the structure and diversity of both algal and bacterial assemblages in biofilm. In our study, shading significantly reduced the prevalence of diatoms known to cause nuisance blooms under nutrient-enriched conditions, and created communities that were more even and diverse. Our algal results support findings e.g. by Hill, Fanta and Roberts (2009), Ghermandi *et al.* (2009) and Bowes *et al.* (2012b) that suggest riparian shading may be an effective tool in controlling biofilm growth rates and managing the effects of eutrophication. Whilst it may not seem practicable to have extensive riparian planting schemes, the need to mitigate climate change might make such schemes more palatable. Recent management practice in the UK had begun to advertise how to create riparian shade where it is absent (Lenane 2012). The obvious advantages that shading has in reducing algal blooms and keeping water temperatures low (Warner and Hendrix 1984; Lenane 2012) seem to make the planting of shading desirable even if it is a longer term project (Lenane 2012). Even if, however, riparian shading presents itself as a useful tool to manage eutrophic streams that experience blooms, more research needs to be conducted to assess if shading becomes ineffective as diatoms adjust to lower light levels (Rier, Stevenson and LaLiberte 2006) or are replaced by species better suited to shade. It is also necessary to investigate if shading has a negative effect on the function of biofilm and on invertebrate grazers.

To understand the observed differences between the communities, it would be useful to investigate any changes in function. This could be an examination to determine if bacterial assemblages in shaded rivers are less equipped to process glycosate, which is produced by periphytic algae, or simply transcriptomic analysis of the whole communities, as transcriptomics would identify if the communities are functionally different. It would also be interesting to test whether the diverse and even communities created by riparian shading prove to be more resilient to stress and resistant to pollution events. In that context, it should be tested what effect different community assemblages have on nutrient cycling and biofilm function. Another question is to what degree changes to biofilm nutrient stoichiometry (Cross *et al.* 2005) cause changes to higher trophic levels. Whilst shading might shift biofilm community structure in such a way that harmful blooms are reduced, it might produce unexpected effects on higher trophic levels in the river.
